# Iris mammillations in Cri-du-chat syndrome^☆^

**DOI:** 10.1016/j.ajoc.2025.102442

**Published:** 2025-09-26

**Authors:** Renée S. Landzberg, Julius T. Oatts, Neel D. Pasricha

**Affiliations:** a School of Medicine, University of California San Francisco, San Francisco, CA, USA; b Division of Ophthalmology, Children's Hospital of Philadelphia, Scheie Eye Institute, Perelman School of Medicine at the University of Pennsylvania, Philadelphia, PA, USA; c Department of Ophthalmology, University of California San Francisco, San Francisco, CA, USA; d Francis I. Proctor Foundation, University of California San Francisco, San Francisco, CA, USA

**Keywords:** Cri-du-chat syndrome, Iris mammillations, *PSEN1* mutation

## 1 Case report

**Fig. 1:**
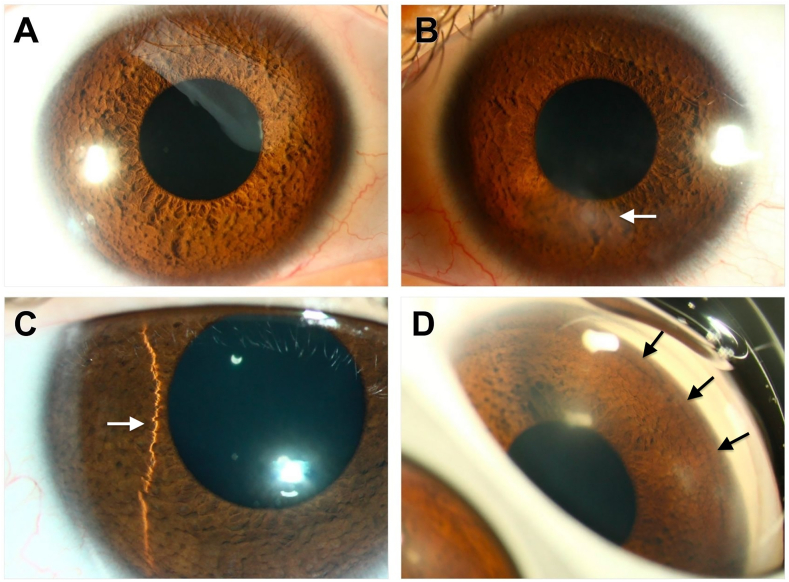
Images from a 14-year-old male with Cri-du-chat syndrome. A/B. Diffuse illumination slit lamp photos of the right (A) and left (B) eyes demonstrating diffuse iris mammillations (IM). Note the cornea is clear on the right eye (A), but the left eye shows faint anterior stromal scarring (arrow) inferior to the visual axis. C. Slit lamp photo using a thin slit beam (arrow) revealing the corrugated appearance of IM. D. Gonioscopic view highlighting elevated IM (arrows) extending to the angle.

A 14-year-old male referred with Cri-du-chat syndrome had characteristic *de novo* deletions of 5p15.33–p15.2 (chr5:132,145–9,670,963; ∼9.54 Mb), classified as pathogenic, and a small duplication of 14q24.2 (chr14:72,612,717–72,775,984; ∼0.163 Mb) involving *presenilin 1* (*PSEN1*), classified as a variant of uncertain significance. These copy number variants were identified by chromosomal microarray analysis using the Agilent 105K oligonucleotide array, performed at the CLIA-certified TPMG Regional Genetics Laboratory. Examination found hypertelorism, exotropia, and myopia, typical of Cri-du-chat, and bilateral iris mammillations (IM), which are small, evenly spaced, hyperpigmented nodules on the anterior iris surface (Fig. 1). Incidental blepharokeratoconjunctivitis was present bilaterally, with inferior anterior corneal stromal scarring and overlying punctate epitheliopathy in the left eye (OS), limiting visual acuity. Best-corrected visual acuity was 20/20 in the right eye (OD) and 20/30 OS. Intraocular pressures (14 mmHg OD and 12 mmHg OS) and funduscopic examination (Supplemental Fig. 1) were normal bilaterally. Corneal tomography showed regular astigmatism of 0.7Dx006 OD and irregular astigmatism of 1.7Dx166 OS. There was corneal steepening with maximum keratometry (Kmax) values of 48.4D OD and 56.6D OS and increased corneal pachymetry of 613 μm OD and 619 μm OS.

## 2 Discussion

We describe bilateral IM in a patient with Cri-du-chat syndrome and a small 14q duplication (including *PSEN1*). Cri-du-chat is a rare genetic syndrome caused by a deletion on chromosome 5, marked by developmental delay, distinctive facial features, and high-pitched cry. Ocular features include hypertelorism, strabismus, refractive error, and optic nerve or retinal pigment abnormalities. IM are typically bilateral and asymptomatic, but may increase intraocular pressure. They have been reported in genetic syndromes including oculodermal melanocytosis and neurofibromatosis type 1.^1^ IM have also been observed in up to 17.9 % of keratoconus patients, with thicker corneas in the IM patients.^2^ Our patient's corneal tomography showed thick corneas and steepening without asymmetric bowtie or other ectatic patterns, and thus no definitive keratoconus. IM have not been reported in association with *PSEN1*; our patient's duplication was small and unlikely of clinical significance, with no reports of anterior segment ocular pathology from *PSEN1* duplications. In *PSEN1* E280A mutation carriers, retinal thinning was observed on optical coherence tomography without anterior segment abnormalities.^3^

## 3 Conclusion

This report of bilateral IM in association with Cri-du-chat syndrome or chromosome 14q duplication underscores the need for further research into the genetic and clinical implications of IM, particularly in rare genetic disorders.

## CRediT authorship contribution statement

**Renée S. Landzberg:** Writing – review & editing, Writing – original draft, Conceptualization. **Julius T. Oatts:** Writing – review & editing, Conceptualization. **Neel D. Pasricha:** Writing – review & editing, Conceptualization.

## Patient consent

Written consent to publish this case has not been obtained. This report does not contain any personal identifying information.

## Authorship

All authors attest that they meet the current ICMJE criteria for Authorship.

## Funding

This study was supported by K08 EY033859 from the National Eye Institute, Career Development Award from the Research to Prevent Blindness, and grant from the All May See Foundation to Dr. Pasricha and UCSF Vision Core Grant (P30 EY002162) and Research to Prevent Blindness Unrestricted Grant to the UCSF, Department of Ophthalmology.

## Declaration of competing interest

The authors declare that they have no known competing financial interests or personal relationships that could have appeared to influence the work reported in this paper.

## Appendix A Supplementary data

Normal bilateral fundus photographs from a 14-year-old male with Cri-du-chat syndrome and iris mammillations OD, right eye; OS, left eye.
