# Supplementary figures and images for: Iris mammillations in Cri-du-chat syndrome
Source: Am J Ophthalmol Case Rep. 2025 Sep 26;40:102442. doi: 10.1016/j.ajoc.2025.102442 (PMC12514350; doi:10.1016/j.ajoc.2025.102442)

OD

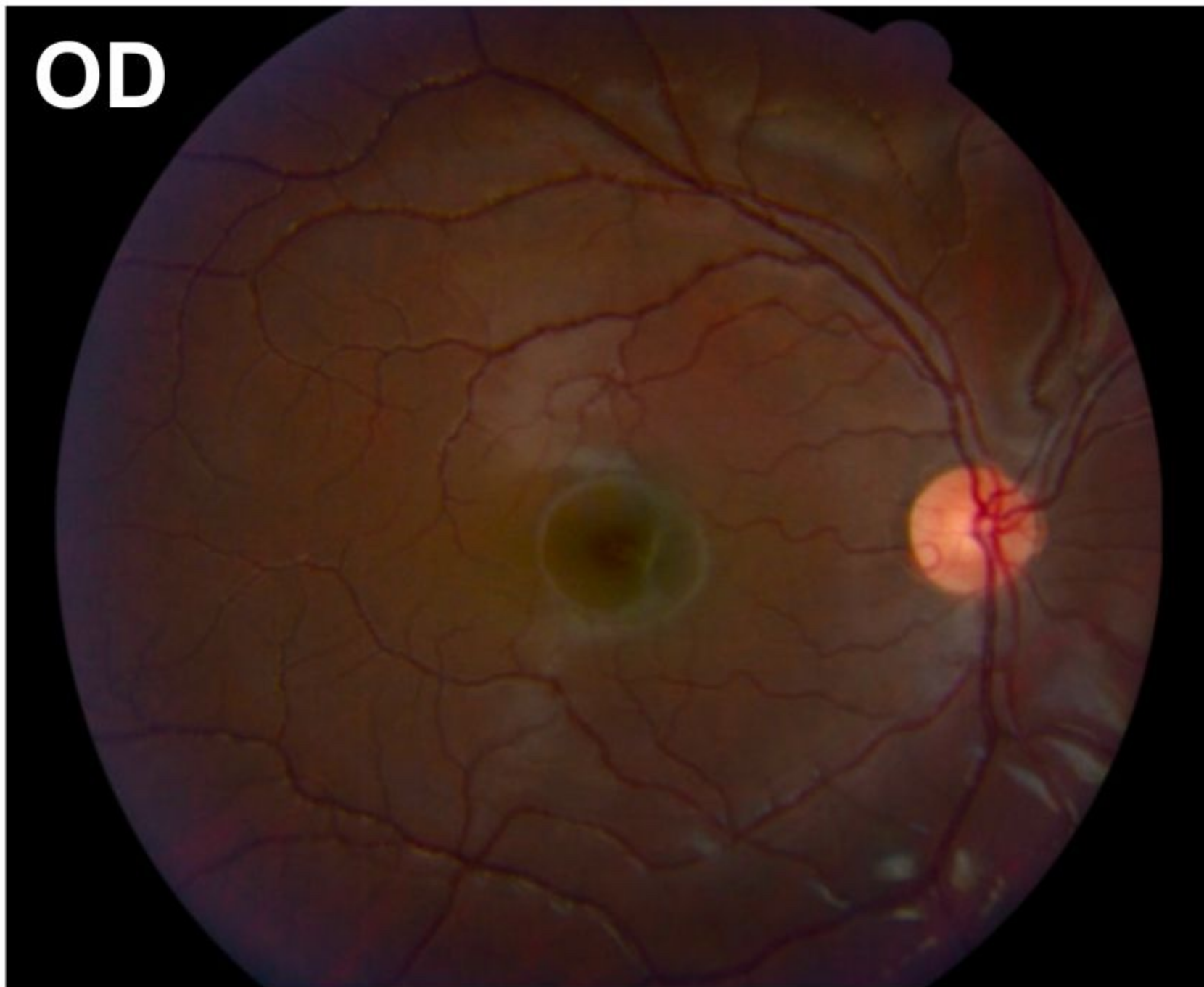

OS

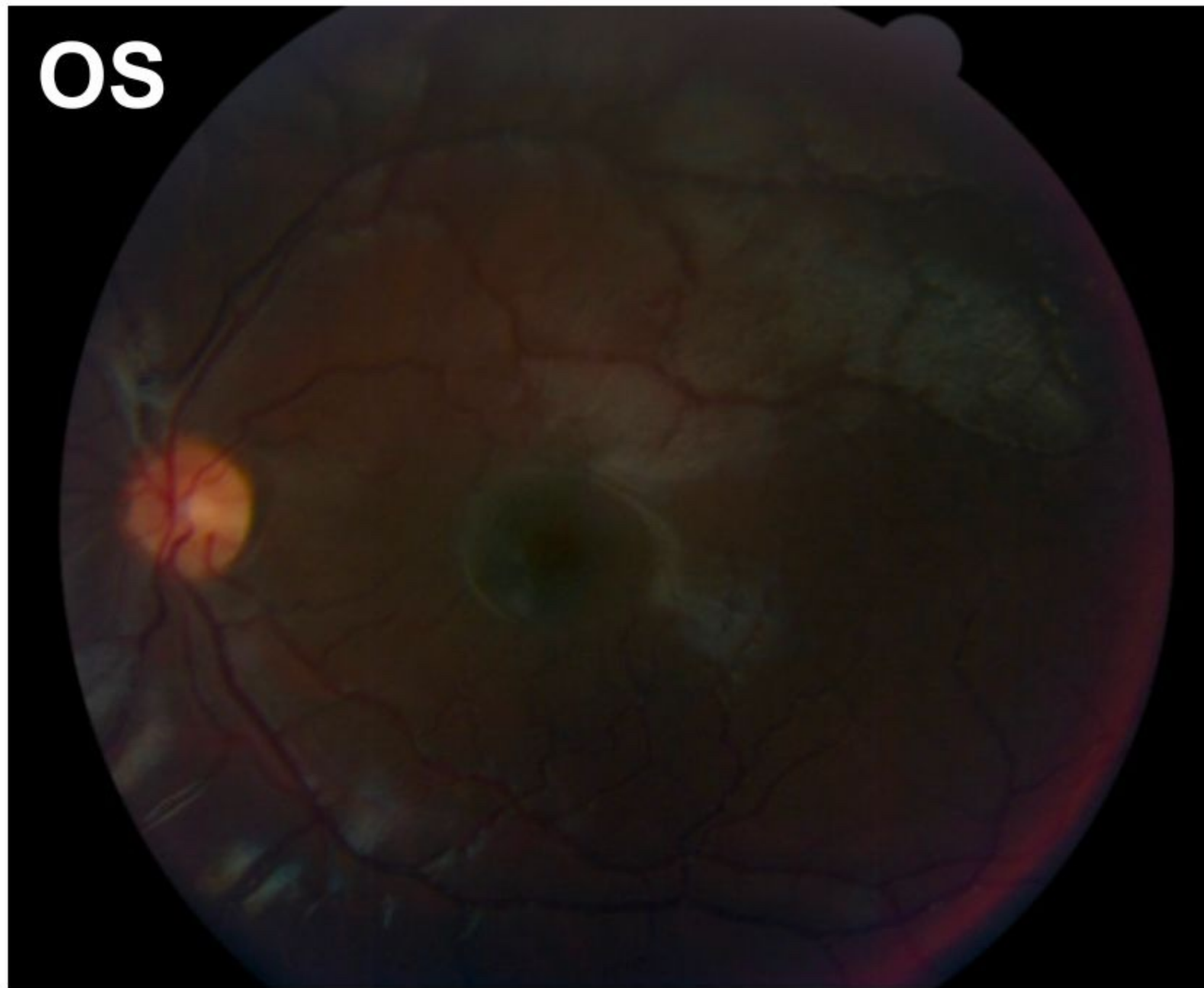

Supplement: Multimedia component 1 [file mmc1.pdf]
